# Aerobic exercise reduces biomarkers related to cardiovascular risk among cleaners: effects of a worksite intervention RCT

**DOI:** 10.1007/s00420-015-1067-5

**Published:** 2015-07-03

**Authors:** Mette Korshøj, Marie Højbjerg Ravn, Andreas Holtermann, Åse Marie Hansen, Peter Krustrup

**Affiliations:** National Research Centre for the Working Environment, Lersø Parkallé 105, 2100 Copenhagen Ø, Denmark; Department of Nutrition, Exercise and Sports, Copenhagen Centre for Team Sport and Health, University of Copenhagen, Nørre Allé 51, Copenhagen N, 2200 Denmark; Institute of Sports Science and Clinical Biomechanics, University of Southern Denmark, Campusvej 55, 5230 Odense M, Denmark; Department of Public Health, University of Copenhagen, Øster Farimagsgade 5, Copenhagen K, 1014 Denmark; Sport and Health Sciences, College of Life and Environmental Sciences, University of Exeter, St. Luke’s Campus, Exeter, UK

**Keywords:** Worksite intervention, Aerobic workload, RCT, Blue-collar workers, Cardiovascular disease, Diurnal measurements, Objective measurements

## Abstract

**Purpose:**

Blue-collar workers have an increased risk of cardiovascular disease. Accordingly, elevated levels of biomarkers related to risk of cardiovascular disease, such as high-sensitive C-reactive protein, have been observed among blue-collar workers. The objective was to examine whether an aerobic exercise worksite intervention changes the level of inflammation biomarkers among cleaners.

**Methods:**

The design was a cluster-randomized controlled trial with 4-month worksite intervention. Before the 116 cleaners aged 18–65 years were randomized, they signed an informed consent form. The reference group (*n* = 59) received lectures, and the aerobic exercise group (*n* = 57) performed worksite aerobic exercise (30 min twice a week). Levels of biomarkers (high-sensitive C-reactive protein, fibrinogen, cholesterol, low- and high-density lipoprotein cholesterol and triglyceride) were collected at baseline and after 4 months. A repeated-measure, multi-adjusted, mixed-model intention-to-treat analysis was applied to compare between-group differences. The study was registered as ISRCTN86682076.

**Results:**

Significant (*p* < 0.05) between-group reductions from baseline to follow-up were found for high-sensitive C-reactive protein (−0.54 ± 0.20 µg/ml; 95 % CI −0.94, −0.14), low-density lipoprotein cholesterol (−0.32 ± 0.11 mmol/L; 95 % CI −0.54, −0.10) and the ratios of LDL/HDL (−0.30 ± 0.08; 95 % CI −0.46, −0.14), and LDL/TC cholesterol (−0.04 ± 0.02; 95 % CI −0.07, −0.01).

**Conclusion:**

This study indicates that an aerobic exercise intervention among cleaners leads to reduced levels of high-sensitive C-reactive protein and low-density lipoprotein cholesterol, and an unaltered level of fibrinogen. The aerobic exercise seems to improve inflammatory levels and lipoprotein profile among cleaners, with no signs of cardiovascular overload.

## Introduction

Blue-collar workers have an increased risk of cardiovascular disease (Li et al. 2013; Zöller et al. 2012). Accordingly, elevated levels of inflammation biomarkers, such as high-sensitive C-reactive protein (hsCRP), have been observed among blue-collar workers (Kittel et al. 2002; Clark et al. 2012). In addition, blue-collar workers are reported to have an unfavourable lipoprotein profile, with increased levels of triglyceride (TG) and total cholesterol (TC), and a high ratio of low-density lipoprotein (LDL cholesterol)/high-density lipoprotein (HDL cholesterol) (Clark et al. 2012; Khanolkar et al. 2012). Interventions targeting the elevated inflammatory levels and unfavourable lipoprotein profile to prevent cardiovascular disease among blue-collar workers are therefore requested.

Many factors, like diet, smoking and physical activity, influence levels of inflammatory biomarkers and the lipoprotein profile both separately and in combinations, as described in previous literature (Grandjean et al. 1996; Pedersen and Saltin 2006). Also, aerobic exercise has been previously shown to effectively reduce levels of inflammation biomarkers (Okita et al. 2004; Loprinzi et al. 2013; Plaisance and Grandjean 2006; Kasapis and Thompson 2005) and thereby risk of cardiovascular disease (Danesh et al. 2005; Kaptoge et al. 2010; de Ferranti and Rifai 2007). This may be explained by the adaptations from aerobic exercise leading to a lowered acceleration of the inflammation in the arterial endothelia initiated by a lowered LDL concentration. Since the hsCRP binds to the LDL, it is thereby also lowered (de Ferranti and Rifai 2007; Kasapis and Thompson 2005; Lusis 2000). Aerobic exercise is therefore recommended to prevent the excessive risk of cardiovascular disease among blue-collar workers (Li et al. 2013; Zöller et al. 2012). However, we are not aware of previous studies that have evaluated the effect of an aerobic exercise worksite intervention on inflammatory biomarkers in a blue-collar population. The worksite is a recommended arena for physical activity interventions (Heath et al. 2012), mainly because it offers opportunities to reach specific high-risk groups exposed to similar risk factors.

Blue-collar workers, such as cleaners, are often exposed to high volumes of occupational physical activity (Steele and Mummery 2003; Søgaard et al. 2006). Although the volume of occupational physical activity is relatively high (Bonjer 1971), the intensity is not sufficiently high to enhance the cardiorespiratory fitness (Korshøj et al. 2013; Ruzic et al. 2003). Therefore, it may be hypothesized that aerobic exercise could enhance the cardiorespiratory fitness among workers with high levels of occupational physical activity. Thus, the initial combination of a high volume of occupational physical activity, limited possibility for recovery and a low level of cardiorespiratory fitness could overload the cardiovascular system (Clays et al. 2014; Holtermann et al. 2012; Krause et al. 2007). An aerobic exercise intervention, increasing the volume and intensity of physical activity, may therefore progress a potential overload of the cardiovascular system (Krause et al. 2007; Armstrong et al. 2015; Schnohr et al. 2015; Lee et al. 2014) and thereby lead to increased levels of inflammation and risk of cardiovascular disease (Danesh et al. 2005; Kaptoge et al. 2010; de Ferranti and Rifai 2007).

Recently, we found a general reduction in risk factors for cardiovascular disease, but also a clinically significant increased systolic blood pressure following a worksite aerobic exercise randomized controlled intervention among cleaners (Korshøj et al. 2015). This finding indicates that aerobic exercise may overload the cardiovascular system of cleaners, possibly due to their high exposure to occupational physical activity (Søgaard et al. 2006; Krüger et al. 1997), limited possibility for recovery and low level of cardiorespiratory fitness (Korshøj et al. 2013; Louhevaara 1999; Ruzic et al. 2003). Thus, the effects on inflammatory levels and lipoprotein profile of aerobic exercise interventions among cleaners are of particular interest for investigation.

The objective was therefore to examine the effects of an aerobic exercise intervention on inflammatory levels and lipoprotein profile among cleaners. The null hypothesis of the study was that the aerobic exercise intervention would not modify the inflammatory levels and lipoprotein profile.

## Methods

### Study design

The study design represents a cluster-randomized controlled trial. The intervention was divided into two phases with different aims. The aim of the first intervention phase, from baseline to 4-month follow-up, was to evaluate the efficacy of the intervention on biomarkers related to cardiovascular risk (Korshøj et al. 2012).

The study was approved by the Danish Data Protection Agency and the Ethics Committee for the regional capital in Denmark (journal number H-2-2011-116) and subsequently conducted in accordance with the Declaration of Helsinki. The study was registered as ISRCTN86682076 in Current Controlled Trials (Current Controlled Trials 2014).

### Recruitment

Recruitment is described in detail in the protocol paper (Korshøj et al. 2012). Briefly described, the recruitment took place by directly contact to the management of cleaning companies. By confirmation of collaboration, all employees were invited to an information meeting. At the information meeting, the cleaners filled in a screening questionnaire that collected background information such as ethnicity, smoking status and job seniority. Additionally, the cleaners were asked whether they wished to participate in the study. All participants signed an informed consent form agreed upon by the Ethics Committee for the regional capital in Denmark. The authors confirm that all ongoing and related trials for this intervention are registered.

### Study population

The study population comprised cleaners performing predominantly cleaning in day-care institutions, offices, hospitals and schools. At company level, the inclusion criteria were more than 50 employed cleaners and the possibility of the cleaners participating in the project activities during paid working hours. Eligibility criteria were: employment as a cleaner for >20 h per week; age 18‒65 years; and the provision of signed and informed consent. Exclusion criteria were pregnancy and fever on the day of testing. Further exclusion criteria for specific physical tests and blood sampling were moderate and severe hypertension (≥160/≥100 mmHg), angina pectoris, cardiac insufficiency and acute myocardial infarction. The sample size was based on a power calculation on the proposed intervention effect (increase by 4 %) on cardiorespiratory fitness (mlO_2_/min/kg) and showed that it would take 52 participants in each of the two intervention groups to show significance at a level of 0.05. The sample size calculations assumed recruitment of 40 % of eligible cleaners and a dropout rate of 30 % during the intervention (Korshøj et al. 2012).

### Randomization

A full description of the randomization is reported elsewhere (Korshøj et al. 2012). The randomization was performed at cluster level, and clusters were set within strata. Each stratum was formed according to the manager to whom the participant reports, and the clusters were balanced in respect of geographical work location, gender, age and job seniority. Participants were randomly assigned to either a reference group or an aerobic exercise group.

### Intervention

Participants assigned to the aerobic exercise group received 30 min of supervised aerobic exercise training twice a week for 16 weeks at an intensity ≥60 % of maximal oxygen consumption. The type of aerobic exercise was tailored to the specific workplace (Korshøj et al. 2012) via an intervention mapping approach (Bartholomew et al. 1998).

The reference group received two lectures of 2-h duration during the 4-month period. The lectures concerned healthy living, and the participants were invited to give suggestions for the lectures. None of the lectures addressed physical activity.

### Data collection

The baseline measurements were conducted in January 2012 at the first company, May 2012 at the second company and August 2012 at the third company. Follow-up measurements were conducted in May/June 2012 at the first company, January/February 2013 at the second company and February 2013 at the third company, corresponding to follow-up periods of approximately 16 weeks at the first company, 27 weeks (including 10 weeks of summer holidays) at the second company and 18 weeks (including 2 weeks of Christmas holidays) at the third company. The data collection consisted of a questionnaire-based interview including items regarding sociodemographics, lifestyle, ethnicity, education level, job seniority, gender, diagnosed illnesses, use of medication, level of physical activity at work and in leisure time (Saltin and Grimby 1968), and rating of perceived exertion during working hours (Borg 1962). Additionally, measurements of anthropometrics, blood pressure, and blood sampling and fixing of the monitors for the diurnal objective measurements of heart rate were conducted.

### Physiological measurements

Height was measured without shoes in an upright standing position (Seca 213, Birmingham, UK). Body weight, body mass index (BMI) and body fat percentage were determined using bioelectrical impedance analysis (TANITA BC-418, USA). Resting blood pressure was measured three times on the left arm after at least 15 min sitting at rest in a quiet room (Omron M6 comfort, Omron, Helsinki, Finland) (O’Brien et al. 2005). The waist was defined as the narrowest point between the lowest rib and the iliac crest (Canoy 2008). Level of cardiorespiratory fitness was estimated by a sub-maximal step test (Aadahl et al. 2013) conducted on a bench of 30 cm height for females and 35 cm for males.

### Diurnal measurements of heart rate

The participants were asked to wear the monitor 24 h a day. The Actiheart heart rate monitor (CamNtech, Cambridge, UK) was worn for 4 days (two working and two non-working days) and was able to measure continuously as it is water resistant and wireless. The participants were asked to keep an activity log of working hours and sleeping time. Actiheart was initialized and data downloaded using the commercial software (version 4.0.98, CamNtech, Cambridge, UK). Actiheart measures electrocardiographic raw signals by inter-beat intervals with a sensitivity of 0.25 mV (The Actiheart web site 2014). Actiheart is validated for measurement of heart rate, heart rate variability and estimations of energy expenditure in the field (Barreira et al. 2009; Assah et al. 2011). The Actiheart monitors were attached to the skin using two ECG electrodes (Ambu blue sensor VL-00-S/25, Ballerup, Copenhagen; Denmark) at either of the two validated positions at the apex of the sternum, orthogonally to the wire axis, with a horizontal wire to the left, or at the manubrium of the sternum, orthogonally to the wire axis, with a horizontal wire to the left (Brage et al. 2005).

### Blood sampling

Non-fasting blood samples were taken during working hours (7 a.m. to 3 p.m.), and no restrictions were imposed with regard to food, caffeine, tobacco and alcohol consumption or exercise prior to the sampling. The blood samples were stored at −20 °C and ethylenediaminetetraacetic acid plasma at −80 °C until analysed within a maximum of 2 years.

High-performance liquid chromatography (HPLC) with a cation exchange column, Mono S HR 5/5 from Pharmacia Biotech AB, Uppsala, Sweden, was used to determine HbA_1c_. The HPLC consisted of a Waters 625 LC system together with a Waters photodiode array detector model 996 and a WISP 717 auto-sampler for automatic injection of the samples. Millennium chromatography software was used to calculate concentrations (Waters Associates Inc., Milford, US). The method for HbA_1c_ has been evaluated by inter-laboratory comparison based on 17 patient samples and found to be linear in the range 4.1‒14.3 % of total haemoglobin and without systematic bias (Garde et al. 2000). Lyphochek Diabetes Control (Calibrator) from BioRad (Anaheim, CA, US) for HbA_1c_ was used to monitor the long-term stability of the method.

HDL cholesterol, LDL cholesterol and TC were analysed using a Cobas MIRA Plus. The determination of HDL cholesterol, LDL cholesterol, TC and TG was carried out by ABX Pentra assays from Triolab (Sollentuna, Sweden). Calculation of LDL/HDL cholesterol ratio and LDL/TC cholesterol ratio was carried out by dividing LDL cholesterol by HDL cholesterol and LDL cholesterol by TC. The analytical methods for measuring TC in serum have been validated (Christensen et al. 1993; Hansen et al. 2007). Commercially available control samples for HDL cholesterol, LDL cholesterol, TC and TG were analysed together with the samples to show equivalence between different runs. Westgard control charts were used to document that the analytical methods remained in analytical and statistical control, i.e. the precision and trueness of the analytical methods remained stable (Westgard et al. 1981).

Fibrinogen was analysed by turbidimetry on Cobas MIRA Plus. We used a high-sensitive ELISA (EU59151), purchased from IBL, International GMBH, Hamburg, Germany, to measure hsCRP. The between-assay variation was 5.8 % at 1.6 µg/ml, and the limit of detection was 0.02 μg/ml.

### Analyses

The primary outcome in this paper is between-group changes in hsCRP, fibrinogen, HDL cholesterol, LDL cholesterol, TC, TG and HbA_1c_ during the 4 months of follow-up. From the diurnal measures, only heart rate measurements with beat error ≤50 % were included (Skotte et al. 2014) in order to only include diurnal measurements of sufficient quality in the statistical analysis. The relative aerobic workload was calculated as HRR (HRR = HR_max_ − SHR) (Karvonen and Vuorimaa 1988), the difference between the estimated maximal heart rate (HR_max_) (Tanaka et al. 2001) and the sleeping heart rate (SHR), defined as the 10th lowest recorded heart rate value during sleep (Brage et al. 2004). The percentage of the HRR was calculated as [%HRR = ((HR during activity − SHR)/HRR) × 100].

### Statistical analyses

The analyses were performed according to the intention-to-treat (ITT) principle, in which all randomized participants are included in the statistical analyses. Missing values were not imputed either for outcome or for covariate variables (White et al. 2012; Twisk et al. 2013). The analyses were performed to evaluate the between- (aerobic exercise compared to reference) and within-group effects on the outcomes of the 4-month intervention period (follow-up baseline). Analyses followed a repeated-measures 2 × 2 mixed-model design. Independent categorical variables (fixed factors) were group (aerobic exercise and reference), measurement time (baseline and 4-month follow-up) and the interaction between groups and measurement time. Participants were entered in the model as a random effect nested in clusters (i.e. to account for the cluster-based randomization). Covariates to be included were chosen based on baseline differences between groups on theoretically considered confounders and their statistical association with the group and measurement time. The following covariates were incrementally taken into account in the analysis (reference value in parenthesis): baseline value of the respective outcome, age, gender (male), BMI, daily use of cholesterol and/or hormone medication (none), smoking status (never smoked and/or currently non-smoking), level of leisure-time physical activity (<2 h per week of light activity) and alcohol consumption. The covariates were included in the mixed model in two models: the model step included adjustment for the baseline value of the respective outcome, while the second model included additional adjustment for age, gender, BMI, use of cholesterol and/or hormone medication, smoking status, alcohol consumption and leisure-time physical activity. The statistical estimates of the outcomes are reported as between-group mean difference ± SE, 95 % confidence interval and level of significance, where a level of ≤0.05 was set as the level of statistical significance.

Sensitivity analysis was performed by applying the same statistical model with the exclusion of participants reporting daily use of cholesterol and/or hormone medication. Additionally adjustment for western/non-western ethnicity was applied to the fully adjusted model 2 in the between-group analysis.

All statistical analyses were conducted using IBM SPSS statistical software (version 21) (Armonk, NY, US) and the SAS statistical software for Windows (version 9.3) (Cary, NC, US).

## Results

### Flow of participants

**Fig. 1:**
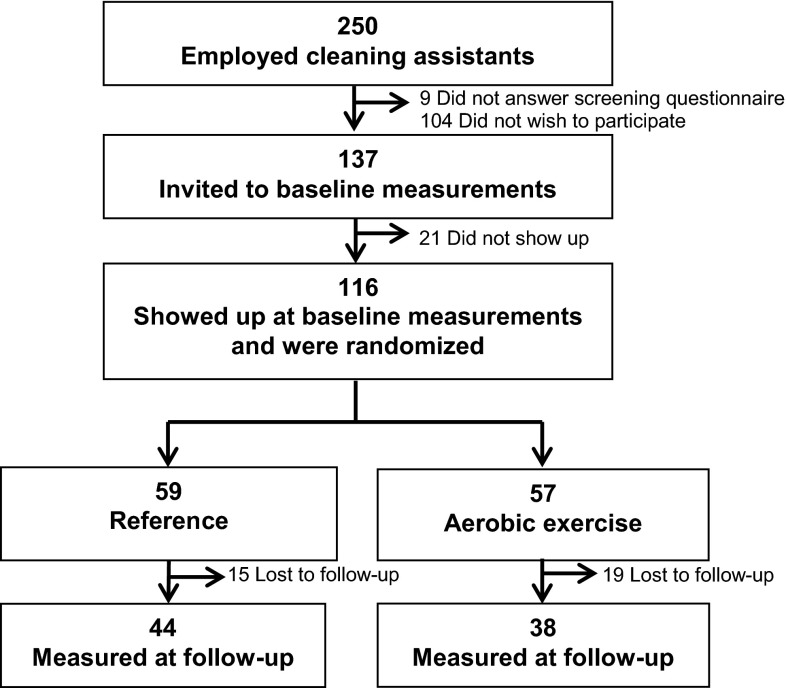
Flow chart for the participants

Of the three companies contacted, all agreed to participate. At these companies, 250 cleaning assistants were introduced to the project. Of those, 137 (45 %) wished to participate and were invited to the baseline measurements. At the baseline measurements, 116 participated and were randomized, with 59 assigned to the reference group and 57 to the aerobic exercise group. Of those participants who were randomized, 34 (29 %) dropped out of the project and were lost to follow-up (15 from the reference group and 19 from the aerobic exercise group) (Fig. 1), (Korshøj et al. 2015).

### Compliance

The real dropout in this study (29 %) was below the expected dropout (30 %) from baseline to follow-up. On average, the participants randomized to the aerobic exercise took part in 51 % of the planned sessions during the intervention period, including a total of five participants with zero adherences. Those participants not lost to follow-up participated in 64 % of the planned sessions, with no zero adherences (Korshøj et al. 2015).

After every fourth week of intervention, heart rate was monitored at three time-points during the aerobic exercise session, yielding an average heart rate of 67 (±13 SD)  % HRR (Korshøj et al. 2015).

### Baseline characteristics of the study population

Table 1 presents the baseline characteristics of the study population. The only significant difference between the aerobic exercise group and the reference group was the length of education, where a higher amount of participants in the reference group had >12 years of education. Additionally, no statistical or numerically significant differences were observed between the randomized population and the population participating either at baseline or at the follow-up measurements (complete to follow-up). Within the aerobic exercise group, the 19 participants (33.3 %) lost to follow-up had different numerical values to the true population on the following parameters: age (2.5 years younger), gender (1.7 % less females), smoking (19.7 % more smokers) and leisure-time physical activity (5.2 % more stated to be active ≥2 h of moderate activity per week). Within the reference group, the 16 participants (25.4 %) lost to follow-up had different numerical values to the true population on the following parameters: age (0.9 years younger), gender (3,0 % less females), smoking (14.6 % more smokers) and leisure-time physical activity (3.1 % more stated to be active ≥2 h of moderate activity per week) (Korshøj et al. 2015).

**Table 1:** Description of randomized study population at baseline measurements (*N* = 116), stratified in the aerobic exercise group (*n* = 57) and reference group (*n* = 59). Mean ± SD is reported

	Randomized population (*N* = 116)			Aerobic exercise (*n* = 57)			Reference (*n* = 59)		
	Mean	SD	*n*	Mean	SD	*n*	Mean	SD	*n*
Age (years)	45.3	8.6		44.9	9.2		45.7	8.1	
Gender (% of females)	75.9		88	75.4		43	76.3		45
Height (m)	1.63	0.09		1.63	0.09		1.62	0.08	
Weight (kg)	70.7	14.1		69.7	12.7		71.7	15.4	
Body mass index (kg/m^2^)	26.7	4.5		26.2	4.0		27.1	4.9	
Systolic blood pressure (mmHg)	122.7	21.7		125.2	25.1		120.3	17.5	
Diastolic blood pressure (mmHg)	82.7	12.6		83.7	14.2		81.7	10.8	
Waist circumference (cm)	87.6	11.1		86.7	11.0		88.4	11.2	
Cardiorespiratory fitness (mlO_2_/min/kg)	24.9	6.6		24.8	5.8		25.0	7.2	
Resting heart rate (beats/min)	71.3	14.8		71.7	10.6		70.5	8.8	
Sleeping heart rate (beats/min)	49.5	5.8		49.2	6.5		49.7	5.1	
Aerobic workload (% of HRR)	30.9	7.2		30.1	6.7		31.7	7.5	
Job seniority (years)	11.9	7.8		12.3	8.7		11.5	6.8	
Current smoker (%)	24.1		28	22.8		13	25.4		15
Education (% with >12 years education)	11.2		13	5.3*		3	16.9*		10
Ethnicity (% non-western)	62.1		72	70.2		40	54.2		32
Daily use of cholesterol and/or hormone medication (%)	13.8		16	12.3		7	15.3		9
Leisure-time physical activity (% <2 h/week light activity or light activity 2–4 h/week)	72.4		84	78.9		45	66.1		39
Physical activity at work (% having standing/walking work including lifts and strenuous physical work)	60.3		70	63.2		36	57.6		34
High-sensitive C-reactive protein (µg/mL)	1.47	1.72		1.39	1.30		1.53	2.05	
Fibrinogen (g/L)	3.22	0.68		3.24	0.69		3.19	0.67	
High-density lipoprotein (mmol/L)	1.53	0.39		1.53	0.36		1.53	0.41	
Low-density lipoprotein (mmol/L)	3.32	1.00		3.50	0.98		3.16	1.00	
Total cholesterol (mmol/L)	5.66	1.25		5.82	1.25		5.50	1.24	
Triglyceride (mmol/L)	1.45	0.77		1.48	0.82		1.42	0.71	
Glycated haemoglobin (%)	5.24	0.65		5.29	0.77		5.20	0.51	
Low-density lipoprotein/high-density lipoprotein ratio	2.28	0.82		2.38	0.76		2.19	0.87	
Low-density lipoprotein/total cholesterol ratio	0.58	0.08		0.60	0.08		0.57	0.08	

Differences between aerobic exercise and reference groups in continuous variables are analysed using a Student’s *t* test, and categorical variables are analysed using a Chi-square test

* Significant between-group difference (*p* ≤ 0.05)

### Between-group intervention effects

Table 2 presents the between-group differences in the intervention-induced change in biomarkers from baseline to follow-up. The aerobic exercise group significantly decreased the level of hsCRP in comparison with the reference group by 33 % (model 1) relative to the overall baseline mean in the randomized population. The fully adjusted model (model 2) showed a between-group difference in hsCRP change corresponding to 37 % in the aerobic exercise group. Non-significant between-group changes in fibrinogen were found in both model 1 and the fully adjusted model (model 2).

**Table 2:** Between-group difference (mean ± SE) from baseline to 4-month follow-up on the outcomes in the randomized population of cleaners (*N* = 116)

	Model 1					Model 2				
	Δ	SE	95 % CI	*p*	*n*	Δ	SE	95 % CI	*p*	*n*
Δ High-sensitive C-reactive protein (µg/mL)	−0.48	0.20	−0.87, −0.09	0.02	102	−0.54	0.20	−0.94, −0.14	<0.01	93
Δ Fibrinogen (g/L)	−0.13	0.09	−0.31, 0.05	0.15	102	−0.13	0.10	−0.33, 0.06	0.18	93
Δ High-density lipoprotein (mmol/L)	<−0.01	0.03	−0.05, 0.05	1.00	102	0.01	0.03	−0.04, 0.06	0.72	93
Δ Low-density lipoprotein (mmol/L)	−0.30	0.11	−0.51, −0.09	<0.01	102	−0.32	0.11	−0.54, −0.10	<0.01	93
Δ Total cholesterol (mmol/L)	−0.21	0.12	−0.44, 0.02	0.08	102	−0.24	0.13	−0.50, 0.01	0.06	93
Δ Triglyceride (mmol/L)	0.23	0.11	0.02, 0.44	0.03	102	0.18	0.10	−0.02, 0.39	0.08	93
Δ Glycated haemoglobin (%)^a^	0.01	0.05	−0.09, 0.10	0.92	102	0.04	0.05	0.06, −0.14	0.40	93
Δ Low-density lipoprotein/high-density lipoprotein ratio	−0.27	0.08	−0.42, −0.12	<0.01	102	−0.30	0.08	−0.46, −0.14	<0.01	93
Δ Low-density lipoprotein/total cholesterol ratio	−0.04	0.02	−0.07, −0.01	0.02	102	−0.04	0.02	−0.07, −0.01	0.03	93

Between-group 95 % confidence interval and level of significance are reported

Results are based on a mixed-model analysis with step-wise entry of covariates in two models

Model 1 is adjusted for the baseline value of the respective outcome

Model 2 is additionally adjusted for age, gender, BMI, daily use of cholesterol and/or hormone medication, smoking, alcohol consumption and leisure-time physical activity

*n* differs between model 1 and model 2 due to missing observations in covariate and/or outcome variables

^a^Due to missing data in the covariates, model 2 did not converge for HbA_1c_, so the estimates for this outcome are not adjusted for alcohol in model 2

No between-group difference was found in the change in HDL cholesterol in either model 1 or the fully adjusted model (model 2). Both model 1 and the fully adjusted model (model 2) showed a significant between-group difference in LDL cholesterol change. In model 1, this difference corresponded to a decreased level of LDL cholesterol for the aerobic exercise group corresponding to 9 % relative to the reference group. In the fully adjusted model (model 2), this difference corresponded to 10 %. Non-significant between-group changes in TC were found in both of the models (models 1 and 2). A significant between-group difference in TG change was seen in model 1. This difference corresponded to an increased level of TG for the aerobic exercise group corresponding to 16 % relative to the reference group. When analysing the level of TG in the fully adjusted model (model 2), no statistical between-group changes were seen. Non-significant between-group changes in HbA_1c_ were found in both of the models (models 1 and 2). Due to missing covariate observations, the fully adjusted model (model 2) did not converge, except when the adjustment for alcohol was not taken into account. When evaluating the between-group difference in the change in LDL/HDL cholesterol ratio, a significant decrease was observed corresponding to 12 % in the aerobic exercise group relative to the reference group (model 1). The fully adjusted model (model 2) revealed a similar difference corresponding to a 13 % decrease for the aerobic exercise group. Both model 1 and the fully adjusted model (model 2) show a significant between-group difference in the change in LDL/TC cholesterol ratio corresponding to a decrease of 7 % for the aerobic exercise group in relation to the reference group.

### Within-group intervention effects

The fully adjusted within-group differences (model 2) from baseline to follow-up are presented in Table 3. The aerobic exercise group significantly decreased the level of hsCRP by 29 %, LDL by 25 %, TC by 5 %, the LDL/TC ratio by 22 % and LDL/HDL ratio by 25 %. Additionally, significant increases were seen for the levels of TG by 25 % and HbA_1c_ by 2 %.

**Table 3:** Within-group difference (mean ± SE) from baseline to 4-month follow-up on the outcomes in the randomized population of cleaners (*N* = 116)

	Aerobic exercise group				Reference group			
	Δ	SE	95 % CI	*p*	Δ	SE	95 % CI	*p*
Δ High-sensitive C-reactive protein (µg/mL)	−0.41	0.19	−0.04, −0.78	0.03	0.17	0.18	−0.18, 0.53	0.34
Δ Fibrinogen (g/L)	0.14	0.09	−0.04, 0.33	0.12	0.28	0.09	0.10, 0.45	<0.01
*Δ* High-density lipoprotein (mmol/L)	−0.03	0.02	−0.08, 0.02	0.23	−0.04	0.02	−0.09, 0.00	0.07
Δ Low-density lipoprotein (mmol/L)	−0.88	0.10	−1.09, −0.68	<0.01	−0.56	0.10	−0.36, −0.75	<0.01
Δ Total cholesterol (mmol/L)	−0.31	0.12	−0.54, −0.07	0.01	−0.03	0.11	−0.25, 0.19	0.78
Δ Triglyceride (mmol/L)	0.37	0.10	0.18, 0.56	<0.01	0.20	0.09	0.02, 0.38	0.03
Δ Glycated haemoglobin (%)^a^	0.11	0.05	0.02, 0.20	0.02	0.07	0.05	−0.02, 0.16	0.11
Δ Low-density lipoprotein/High-density lipoprotein ratio	−0.59	0.08	−0.74, −0.44	<0.01	−0.30	0.07	−0.44, −0.15	<0.01
Δ Low-density lipoprotein/Total cholesterol ratio	−0.13	0.02	−0.17, −0.10	<0.01	−0.09	0.02	−0.06, −0.12	<0.01

Within-group 95 % confidence interval and level of significance are reported

Results are based on a mixed-model analysis with step-wise entry of covariates in two models. Only the fully adjusted model is presented here

Model 2 is adjusted for baseline value of the respective outcome, age, sex, BMI, daily use of cholesterol and or hormone medication, smoking, alcohol and leisure-time physical activity

^a^Due to missing data in the covariates, model 2 did not converge for HbA_1c_, so the estimates for this outcome are not adjusted for alcohol in model 2

Within the reference group, the fully adjusted within-group differences (model 2) from baseline to follow-up showed significantly decreased levels of LDL by 37 %, LDL/HDL ratio by 14 % and LDL/TC ratio by 16 %. Additionally, significant increases were seen for the levels of fibrinogen by 9 % and TG by 14 %.

### Sensitivity analysis

After excluding participants reporting a daily use of cholesterol and/or hormone medication (*n* = 16), the analysis yields similar between-group differences from baseline to follow-up in the fully adjusted model (model 2), except for the LDL/TC ratio, where the sensitivity analysis did not reach statistical significance. In this population without daily use of cholesterol and/or hormone medication (*n* = 100), the within-group changes from baseline to follow-up are similar to the results in the randomized population.

By further adjusting for baseline occupational physical activity, similar between-group results are obtained. Also, when additionally adjusting for ethnicity (western/non-western), similar between-group results are obtained.

## Discussion

### Summary of results

The main results of this study are that the aerobic exercise group, compared with the reference group, significantly decreased levels of hsCRP, LDL cholesterol, and LDL/HDL and LDL/TC cholesterol ratios after the 4-month intervention period. No between-group differences were observed for fibrinogen, HDL cholesterol and HbA_1c_. Thus, aerobic exercise seems to improve inflammatory levels and lipoprotein profile among cleaners, with no signs of cardiovascular overload.

### Change in biomarkers

Between-group comparisons including all randomized participants show a 37 % decrease in hsCRP in the aerobic exercise group. A difference of this magnitude can be considered clinically relevant, as this lowered level would be expected to decrease the risk of cardiovascular death (Kaptoge et al. 2010). Based on this reduced level of hsCRP, the null hypothesis considering hsCRP can be falsified. No between-group differences in the level of fibrinogen following the intervention were found.

Previous studies indicate that moderate-to-high-intensity aerobic exercise decreases the levels of inflammatory biomarkers such as hsCRP and fibrinogen (Okita et al. 2004; Loprinzi et al. 2013; Plaisance and Grandjean 2006; Kasapis and Thompson 2005) when applied along with sufficient recovery (Knez et al. 2006). The introduction of the aerobic exercise intervention was therefore expected to reduce the levels of inflammatory biomarkers (Okita et al. 2004; Loprinzi et al. 2013; Plaisance and Grandjean 2006; Kasapis and Thompson 2005), as shown for hsCRP. These studies (Okita et al. 2004; Loprinzi et al. 2013; Plaisance and Grandjean 2006; Kasapis and Thompson 2005) were conducted as studies of healthy populations and not of blue-collar workers in particular. This study finds similar effects of aerobic exercise to the previous studies on hsCRP, but not fibrinogen, and not indicating any cardiovascular overload.

The between-group differences following the intervention showed a significantly reduced level of LDL cholesterol for the aerobic exercise group of 10 %. This reduction is considered clinically significant, as it corresponds to a decreased risk of cardiovascular events (Delahoy et al. 2009). People exposed to an energy expenditure above 2200 kcal/week do not seem to benefit from additional physical activity energy expenditure with respect to levels of lipoprotein (Durstine et al. 2001; Hurley et al. 1988). Cleaners are generally exposed to an energy expenditure above 2200 kcal/week (Krüger et al. 1997), and consequently, the reduced level of LDL cholesterol was not expected. Our study therefore indicates that additional moderate-to-high-intensity aerobic exercise may benefit the level of LDL cholesterol among cleaners, in spite of their baseline level of energy expenditure. Accordingly, the null hypothesis regarding no change in levels of lipoproteins between groups is falsified for LDL cholesterol and for LDL/HDL and LDL/TC cholesterol ratios. The initial energy expenditure may be one explanation of why the other measured lipoproteins did not show significant between-group differences following the intervention in the fully adjusted model (model 2, Table 2).

As for lipoproteins, the level of HbA_1c_ is affected by the general level of energy expenditure and not the intensity of physical activity (Umpierre et al. 2011). Accordingly, the analysis did not show significant between-group differences in the level of HbA_1c_ following the intervention (model 2, Table 2).

### Practical implications

Improved knowledge of how to reduce cardiovascular risk among blue-collar workers is needed (Li et al. 2013). Cleaners have increased risk of cardiovascular disease (Zöller et al. 2012; Sjögren et al. 2003) and therefore constitute a relevant study population for preventive interventions, such as this study. This study showed that just 30 min of worksite aerobic exercise at a moderate-to-high intensity (≥60 % of relative heart rate) twice a week for 4 months during paid working hours (Korshøj et al. 2012) reduced levels of hsCRP and LDL cholesterol, and LDL/HDL and LDL/TC cholesterol ratios. Based on the biomarkers of inflammation, this study shows no signs of the possible cardiovascular overload among workers with high occupational physical activity. Because of the overall reduction in risk factors for cardiovascular disease, but also an increased systolic blood pressure (Korshøj et al. 2015), the effects of aerobic exercise among cleaners seem beneficial, but special attention to blood pressure is encouraged.

### Methodological considerations—strengths and limitations

The cluster-randomized controlled trial design and the ITT analysis (Detry and Lewis 2014) represent strengths, reducing possible bias and contamination. Additionally, the mixed model enables use of information from all observations without imputing missing observations (White et al. 2012; Twisk et al. 2013). In terms of the intervention, the modified intervention mapping approach (Korshøj et al. 2012) is a strong feature, since the intervention is tailored specifically to the individual needs and wishes of the participating workplace and employees.

The generalizability of the findings is limited by the convenience sampling of only three companies in the area of Copenhagen, Denmark. The following significant (*p* < 0.05) differences were seen between those wanting to participate (consenters) and those not wanting to participate (non-consenters): the consenters had less job seniority and were more frequently born outside Denmark than the non-consenters. No differences were seen in diagnosed illnesses, level of occupational or leisure-time physical activity, gender, age, height, weight and job seniority. Overall, we consider the findings of the study to be generalizable. The cluster randomization was not accounted for in the sample size calculations. Another limitation of the study’s results is missing observations. Observations are missing at random, which is accounted for by applying the mixed model, but also not at random due to exclusions from cardiorespiratory fitness testing. The number of participants dropping out between baseline and follow-up is not equally distributed between the intervention groups. In the aerobic exercise group, 19 participants were lost to follow-up, while in the reference group 15 participants were lost to follow-up (Korshøj et al. 2015). Given the small difference in the amount of participants lost to follow-up in the groups, we do not expect it to impact the results. The group lost to follow-up had a lipoprotein profile related to a higher cardiovascular risk at baseline than the population with completers, but more favourable levels of inflammation biomarkers. Those lost to follow-up would possibly therefore respond to the intervention differently to those not lost to follow-up, which means that the presented results could be skewed due to selection bias.

At the time of blood sampling, the participants were not fasting, and alcohol, tobacco and food consumption were not recorded before sampling, which could affect the levels of measured biomarkers and consequently the results. The half-life of HbA_1c_ (120 days) may be having an effect in this study, since the follow-up period of 4 months may be too short for evaluating the intervention effects. Changes in biomarkers reflect normal cyclical biological variations (e.g. diurnal and seasonal variations) (Hansen et al. 2007; Garde et al. 2000). Given that there was both a reference and an aerobic exercise group at each of the companies and that the baseline measurements were conducted at the three companies in January, August and September, we do not expect any bias from seasonal variation.

Additionally, we do not know whether the results are biased by psychosocial work environment due to lack of control by these factors. However, the cluster randomization is likely to minimize such potential bias.

## Conclusion

This cluster-randomized study indicates that a worksite aerobic exercise intervention among cleaners leads to decreased levels of hsCRP and LDL cholesterol, and LDL/HDL and LDL/TC cholesterol ratios. Consequently, the aerobic exercise seems to improve inflammatory levels and lipoprotein profile among cleaners, with no signs of cardiovascular overload. Additional studies are needed to replicate these results in other blue-collar populations and to be able to make recommendations regarding physical exercise for workers with high levels of occupational physical activity.
